# The Transformative Effect of Electron Diffraction

**DOI:** 10.1063/4.0000849

**Published:** 2025-10-27

**Authors:** Pierre Le Magueres, Joseph D Ferrara, Robert Bücker

**Affiliations:** 1 Rigaku Americas, The Woodlands, TX, USA; 2 Rigaku europe SE, Neu-Isenburg, Hesse, Germany

## Abstract

Crystal structures are critical to unambiguously determine a compound’s identity, absolute configuration, molecular connectivity and crystal form, as well as whether polymorphs coexist or how the solid-state structure may explain a compound’s behavior and physical properties. X-ray crystallography is the most commonly used method to obtain crystal structures, but it can be limiting if samples with suitable crystallinity, dimensions and quantity are lacking.

Electron diffraction offers a new solution by providing high-resolution structural insights from submicron-size samples. Scratching the bottom of a beaker to gather nanograms of a crystalline powder may suffice for electron diffraction study. In other words, electron diffraction allows for single- crystal crystallography to be performed on powder samples. This allows for solving crystal structures that were previously unsolvable, but also screening crystallization conditions even when trials yield only a small amount of precipitate and automating polymorphs screening over hundreds of nanocrystals spread on a TEM grid.

In this presentation, we will review the fundamentals of electron diffraction, the best practices for sample preparation and demonstrate how it is reshaping research in fields such as pharmaceuticals, metal-organic frameworks, synthetic chemistry and materials science.

